# Examining the Claim that 80–90% of Suicide Cases Had Depression

**DOI:** 10.3389/fpubh.2013.00062

**Published:** 2013-12-06

**Authors:** Said Shahtahmasebi

**Affiliations:** ^1^Centre for Health and Social Practice, Wintec, Hamilton, New Zealand; ^2^The Good Life Research Centre Trust, Christchurch, New Zealand; ^3^Voluntary Faculty, University of Kentucky, Lexington, KY, USA

**Keywords:** anti-depressant, psychological autopsy, selection bias, suicide prevention, mental illness

There is a belief amongst the medical profession and the public that depression causes suicide. When this belief is challenged the medical profession quote that 80–90% of suicides had depression. Where did this estimate come from and what is it based on? If it is true, why then we have not observed a drop in suicide rate given that prescriptions for anti-depressants have increased sharply over the last 12–15 years? For example the New Zealand government’s documents report that prescriptions for anti-depressants had doubled by 2006, and a more recent document reports that it has doubled again since 2006, i.e., the rate has quadrupled over the last 12 years. Yet, suicide rates have maintained an increasing trend over the same period (1).

When I was at primary school and subsequently high school my teachers used to tell me and other vertically challenged students to play basketball so we would become tall. Well, because I was young and because society at that time had faith in authority, in particular, teachers and doctors, I believed them. I tried to get on basketball teams but no one would have me because I was too short!

This is known as “selection bias” where it is not the game that causes children to grow tall, rather, it is the nature of the game that seeks out the selection of tall people.

Unfortunately, this kind of bias, plus measurement error and other statistical bias are frequently allowed into studies of topics related to human behavior including suicide but most studies fail to account for selection bias.

It is estimated that depression is common in the general population. In New Zealand it is estimated that roughly one in six people will suffer from serious depression at some time in their life^1^. In other words, at any one point in time in New Zealand we can expect over half a million people to be suffering from depression. If the 80–90% probability is applied to the population of those with depression in New Zealand then we should expect thousands and thousands of suicides every year. Even if we apply the inverse of 90% (i.e., 0.01) to the population of those suffering from depression we could expect over 5500 cases per year. Yet, on average the number of suicides in New Zealand’s is 540 per year which is 540 too many. However, this suggests a crude suicide risk of 0.00014 (or roughly 14 per 100,000 based on a total population of 4 million). The main depression website (see text footnote 1) which is also part of the New Zealand Government’s suicide prevention strategy states that depression increases the risk of suicide 20-fold. Again a crude calculation suggests (0.00014 × 20 × 500,000 =) 1400 expected suicides per year. Tackling depression has been central to the New Zealand Government’s suicide prevention strategy with the launch of www.depression.org.nz about a decade ago and the quadrupling of anti-depressant prescriptions. Over the same period suicide rates have maintained an upward trend.

Similarly, in Australia national surveys show that 20% of the general population experience significant mental problems each year and the overall suicide rate is about 10 per 100,000. Thus the likelihood of a person with a mental illness taking their own life is low. It is higher for some mental disorders such as bipolar disorder and schizophrenia than others but it also high for those with alcohol and drug problems and addiction, chronic pain, debilitating conditions, and so on (2).

So where does the figure of 80–90% of suicide cases having depression come from?

Some argue that this figure is based on reviewing suicide cases using medical records. The problem with this argument is that if 90% of suicide cases’ medical records show that these cases had been diagnosed with depression, then they must have received treatment for it, so the question arises why then did they go on to complete suicide? Depression, we are told, is treatable for some and can be managed for others. If depression is the cause of suicide then treating the root of suicide should have prevented the 80–90% of cases from committing suicide.

Current estimates suggest that between two-thirds and three-quarters of all suicides do not come into contact with psychiatric services (3, 4). Of the remainder who do have a psychiatric record not all have been diagnosed with depression. For example, in the UK, following a confidential inquiry into homicides and suicides by mentally ill people – data from medical and hospital records were collected on all suicide cases who had been through a community and mental health Trust in the UK (4). The findings from the confidential inquiry revealed that 33% had no diagnosis, 17% had depression either as a diagnosis or mentioned in their hospital notes, followed by 12.5% with schizophrenia, 8 and 6% alcoholism, and personality disorder respectively. Furthermore, the cases from the hospital formed only about one-third of all completed suicides, i.e., two-thirds of all suicides had had no contact with psychiatric services and were successful in their first attempt. Interestingly, for 46% of cases the reason for coming into contact with psychiatric services was due to previous attempts – yet the individuals still completed suicide.

The problem with suicide research is that death only happens once and we have no access to the person who can provide information about their process of decision making: why they chose death instead of life. So we don’t know anything about the two-thirds of suicide cases with no records.

Thus we are still none the wiser as to where the figure that 80–90% of all suicides had depression has come from?

Could it be a case of the old belief or assumption “you must be out of your mind to kill yourself” that has somehow become accepted? The notion that suicide is caused by depression is so strongly established in the mindset that even educated health professionals refuse to question the evidence and thus try to fit every suicide into this model.

For example, a GP giving evidence at a coroner’s inquest in New Zealand in 2005 stated: “I am desperately sad we had no insight into his mental health problem and so were not able to prevent this tragedy.” Why should the GP automatically assume that the young adolescent had a “mental health problem” as it had been reported that the young adolescent was a happy and popular person with no sign of any health problems and no evidence of mental ill-health? In the GP’s mind, selection bias dictated that the young individual must have had mental problems and depression to commit suicide, i.e., we force suicide to fit into the model.

Another example is the case of an Australian celebrity^2^ who following treatment for depression (was prescribed anti-depressants) and was making future plans committed suicide. After the event (suicide) occurred, the psychiatrist’s explanation was that cases with deep depression are good at hiding their feelings and intentions. Once again, in the psychiatrist’s mind, selection bias dictated that nothing other than depression, in this case deep depression (because the case had earlier been treated for depression), could have caused her suicide.

Therefore, the influence from selection bias is automatic. In other words, the public and health professionals automatically assume the presence of depression and mental illness after the event of suicide.

It is surprising that governments are happy to fund researchers to seek information about the mental status of suicide cases from third parties (i.e., family and friends), e.g., the Canterbury Suicide Project (5). These types of suicide research (psychological autopsies) are flawed theoretically, methodologically, and analytically leading to erroneous results and mis-conclusions.

Therefore, it is of no surprise that psychological autopsies have concluded mental illness and depression as the main cause of suicide and 80–90% of suicide cases had had depression, e.g., see Ref. (6).

Clearly, not much reliance can be placed on the results from psychological autopsies, yet, as mentioned earlier, tackling depression is central to the Government’s suicide prevention strategy.

For this strategy to work there has to be a real link between depression and suicide. For the reasons explained above, and the fact that the main informants (suicide cases) can no longer provide objective information, there is no statistical evidence to support the conclusion that depression leads to suicide. If there was a link between depression and suicide a big drop in suicide rates would have followed the Government’s suicide prevention strategy.

Because there has not been a reduction in the suicide rate, any future decrease will be the artifact of the cyclic pattern in suicide rates over time. No doubt, the authorities, as they have done in the past, will claim it as the result of their strategies and continue to provide more of the same but at a much higher cost.

Yes, some suicide victims may have had depression, but these cases form a very small proportion of the population with depression, and not every depressed person kill themselves; some people who experience failure in their lives may kill themselves, but so do successful people; some unemployed people may kill themselves, but so do employed people; etc.

Poor government policies based on poor research and inappropriate information has created a vicious circle. In other words, with a government and health profession focus on a mental illness/depression model will continue to provide more of the same prevention/intervention each year at a much higher cost in terms of lives lost and resources – all because it must be nothing else but depression. As a result we do not know anything about suicide and hence we cannot prevent it. In the meantime, at least a proportion of suicide cases will die needlessly because of our obsession with mental illness and our refusal to address and understand suicide.

## Footnotes

^1^http://www.depression.org.nz/depression

^2^http://www.stuff.co.nz/4262547a1860.html
